# RNA Profiling of Brain Microvessels Reveals Altered Morphology and Signaling in a Mouse Model of Alzheimer’s Disease

**DOI:** 10.21203/rs.3.rs-4178404/v1

**Published:** 2024-04-12

**Authors:** Oandy Naranjo, Olivia M Osborne, Silvia Torices, Sarah Schmidlin, Destiny Tiburcio, Minseon Park, Michal Toborek

**Affiliations:** University of Miami Miller School of Medicine; University of Miami Miller School of Medicine; University of Miami Miller School of Medicine; University of Miami Miller School of Medicine; University of Miami Miller School of Medicine; University of Miami Miller School of Medicine; University of Miami Miller School of Medicine

**Keywords:** Blood-brain barrier, microvessels, pericytes, Alzheimer’s disease, RNAscope, endothelial cells

## Abstract

Disruptions in pericyte and endothelial cell expression can compromise the integrity of the blood-brain barrier (BBB), leading to neurovascular dysfunction and the development of neurological disorders. However, the study of microvessel RNAs has been limited to tissue homogenates, with spatial visualization only available for protein targets. We introduce an innovative microvessel isolation technique that is RNA-friendly for the purpose of coupling with RNAscope analysis. RNA-friendly microvessel isolation combined with RNAscope analysis enables the visualization of cell-specific RNA within the spatial and histological context of the BBB. Using this approach, we have gained valuable insights into the structural and functional differences associated with the microvessels of 5XFAD mice, a mouse model of Alzheimer’s disease (AD). RNAscope analysis revealed a decrease in pericytes from microvessels isolated from 5XFAD mice in comparison to wild-type mice. Additionally, the microvessels of 5XFAD mice exhibited an increase in TYROBP mRNA expression. These findings significantly advance our understanding of neurovascular interactions and hold great promise for guiding the development of targeted therapeutic interventions. This innovative approach enables visualization of cell RNA while preserving the spatial and histological context of the BBB, shedding light on the mechanisms underlying neurovascular unit communication.

## Introduction

The blood-brain barrier (BBB) plays a vital role in maintaining the proper functioning of the central nervous system (CNS). Serving as a highly selective barrier, the BBB orchestrates the exchange of essential molecules between the blood and the CNS while shielding against intruders. Dysfunction or disruption of the BBB can have severe neurological consequences, ranging from increased intracranial pressure to neuronal dysfunction and degeneration. Consequently, gaining a deep understanding of the mechanisms that sustain BBB integrity is vital for diagnosing and treating the root causes of neurovascular diseases like stroke and Alzheimer’s disease (AD).

Since its initial publication in 1969^1^, the isolation of the BBB has been an important methodology in CNS research. Microvessel isolation has emerged as a versatile experimental model for investigating the dynamics of the BBB and the neurovascular unit. The brain microvessels consist of endothelial cells, pericytes, astrocytic foot processes, and neurons, forming a dynamic composition. Through a streamlined procedure lasting approximately three hours, microvessels can be isolated from fresh and frozen brain samples. Traditionally, investigations into the BBB have primarily focused on cellular and molecular aspects, leaving a critical frontier unexplored—the visualization of RNA within microvessels. In this study, we present a novel method for implementing RNA studies on isolated microvessels: the microvessel RNAscope. Our paper aims to shed light on this innovative visual method that highly preserves and selectively visualizes RNAs involved in BBB function and disease.

One area of research that can significantly benefit from microvessel RNAscope is Alzheimer’s disease (AD). AD is a devastating condition characterized by progressive neurological degeneration and the abnormal accumulation of amyloid proteins in the central nervous system (CNS). Neuroimaging studies conducted on individuals in the early stages of AD have revealed BBB breakdown in critical areas such as the hippocampus, as well as multiple gray and white matter regions, often occurring before the onset of dementia^2^. In fact, more than 20 independent postmortem human studies have provided evidence of brain capillary leakage, pericyte and endothelial cell degeneration, loss of BBB tight junctions, and red blood cell extravasation^3^. While various studies emphasize the significance of the BBB in the development of AD, the specific mechanisms underlying its involvement remain unexplored. Herein, we use our novel microvessel isolation technique to study neurovascular unit morphology and signaling with aged 5XFAD mice serving as a model of AD^4^.

## Results

### Microvessel isolation technique for downstream RNAscope analysis

**Figure 1:**
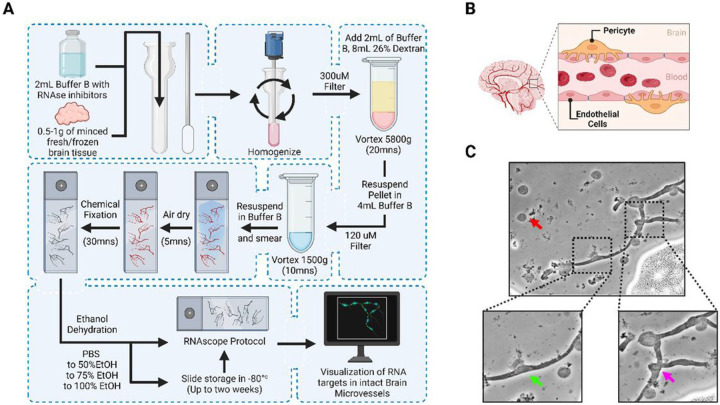
RNAscope Friendly Microvessel Extraction Overview. (A) Schematic overview of the microvascular isolation protocol and RNAscope preparation. (B) Schematic representation of brain microvessel physiology with pericytes and endothelial cells. (C) Light microscopy image of brain microvessels extracted from mouse brain tissue. The arrows emphasize the differences between cellular debris (red arrow), endothelial cell nuclei (green arrow), and pericyte cell nuclei (magenta arrow). Endothelial cells and pericytes appear in a classic “bump on a log” pattern.

A frozen brain hemisphere from wild type (WT) or 5xFAD mice was digested with RNAse inhibitors and buffer before applying to a homogenizer apparatus, followed by filtering through a 300 mm filter. The tissue was separated by centrifugation with 26% dextran solution and the subsequent pellet was filtered through a 120 mm filter before being resuspended and smeared across slides (Figure 1A). After slides were prepped, they went through a chemical fixation that is specific for protecting the viability of the mRNA on the slides. Then the standard RNAscope protocol was followed. Visualization of BBB microvessels independent of other brain cells provides a clear way to assess integrity and function, especially between the endothelial cells (ECs) and pericyte cells (PCs). The dynamic interplay of these two cell types ensures precise control of cerebral blood flow and safeguards the delicate environment of the brain (Figure 1B). Efficient isolation of the microvessels is confirmed with a light microscope that can quickly assess the morphology of the sample. To do this, 15 μL of the resuspended sample were pipetted onto a glass slide and smeared for visualization. Visualization of microvessels on a light microscope confirmed a successful procedure and the expected morphology of vessels before continuing with staining protocol (Figure 1C). We identified normal cellular debris on the slide (Figure 1C, red arrow). Also, we confirmed the presence of microvascular endothelial cells (ECs) by their cell nuclei (Figure 1C, black arrow) and pericyte morphology by its bump on a log morphology (Figure 1C, blue arrow).

### Validity and viability of mRNA targets in isolated microvessels

**Figure 2:**
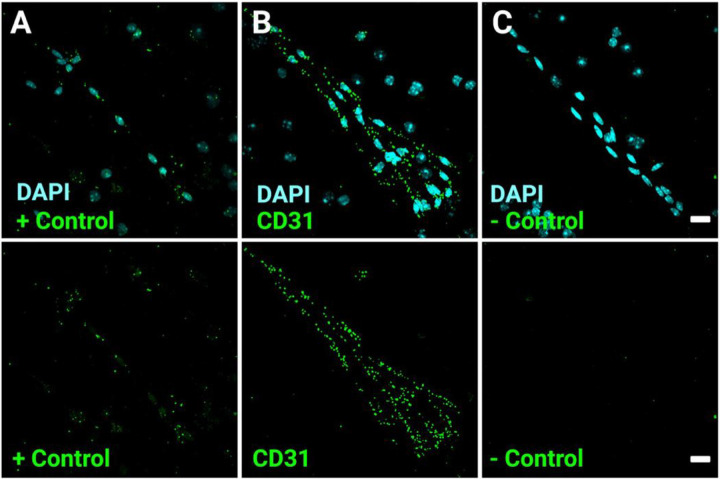
Visualization of microvascular fragments of the blood-brain barrier via CD31 mRNA. (A-C) Confocal imaging of microvessels isolated from wild type mice. DAPI is used to visualize nuclei along with several probes for validation and testing of overall RNA quality. (A) (Upper Panel) Merged image of DAPI in cyan and positive Control Probe (+ Control) in green, (A) (Lower Panel) Isolated image of + control probe in green. (B) (Upper Panel) Merged image of DAPI in cyan and CD31 Probe in green, (B) (Lower Panel) Isolated image of positive CD31 probe in green. (C) (Upper Panel) Merged image of DAPI in cyan and negative control probe (− Control) in green, (B) (Lower Panel) Isolated image of positive - Control probe in green. Scale bars, 20 μm.

Following a successful microvessel isolation, we first assessed the presence of high-quality RNA preserved in the microvessels of wild type mice that could be utilized for downstream visual analysis. A universal marker to confirm the presence of BBB endothelium is CD31^5^. Therefore, smeared glass slides were prepped using our specialized RNAscope protocol for preservation of RNA and probed for CD31 RNA as well as DAPI (Figure 2). To confirm that our RNA probes were specific to viable RNA within the microvessels, we utilized a positive and negative control probe (Figure 2A, C.

Polr2A gene is ubiquitously expressed in all cells and was used as a low copy positive control, this way we can confirm the presence of quality RNA after our microvessel isolation procedure (Figure 2B). Conversely, we used a DapB negative control probe to ensure no background related staining and to confirm the specificity of our overall probe of interest for binding to the correct RNA target.

### Morphology of PDGRFB mRNA on the BBB is markedly altered in 5XFAD mice

**Figure 3:**
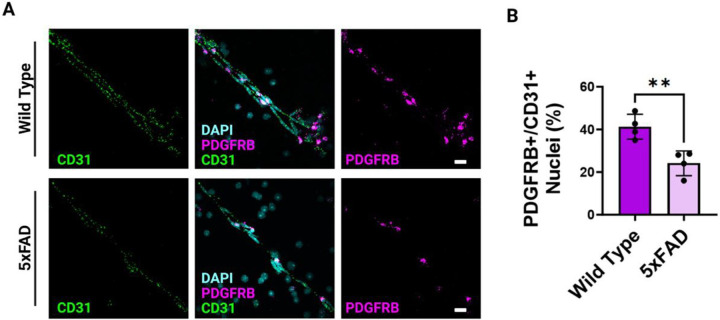
Visualization of PDGRβ mRNA in microvessels isolated from 5XFAD mice. (A) The top panel is representative confocal imaging of microvessels after RNAscope isolated from wild type mice. The bottom panel is representative confocal imaging of microvessels after RNAscope isolated from 5XFAD mice. DAPI is visualized in cyan, CD31 mRNA is visualized in green, and PDGRβ mRNA is visualized in magenta, scale bars, 20 μm. (B) Quantification of PDGRβ mRNA positive nuclei as a percent fraction of total CD31 mRNA positive nuclei. Graph indicates the mean ± SD from 4 independent experiments. *p < 0.05; **p < 0.01; ***p < 0.001; ****p < 0.0001, n=4.

Pericytes are essential to the proper maintenance of the BBB; recent studies in AD research reveal a loss or dysfunction of pericytes at the BBB^6^. Using probes for PDGFRβ and CD31 mRNA, well-known markers for pericytes and endothelial cells respectively^5^, we visualize the BBB of wild type (Fig. 3A, upper panel) and 5XFAD genotypes (Fig. 3A, Lower Panel). Following visualization, we quantified the number of cells positive for PDGRFB as a ratio to cells positive for CD31 and observed a marked decrease in the levels of PDGRβ expression in the 5xFAD mouse compared to control. Wild type mice exhibit around a 1:1 ratio of PDGRβ positive nuclei to CD31 positive nuclei, whereas 5XFAD exhibit around a 1:3 ratio of PDGRβ positive nuclei to CD31 positive nuclei. Results were quantified in multiple microvessels across several aged wild type and 5XFAD mice (Fig. 3B).

### Alzheimer’s disease exacerbates mRNA levels of TYROBP in the endothelium

**Figure 4:**
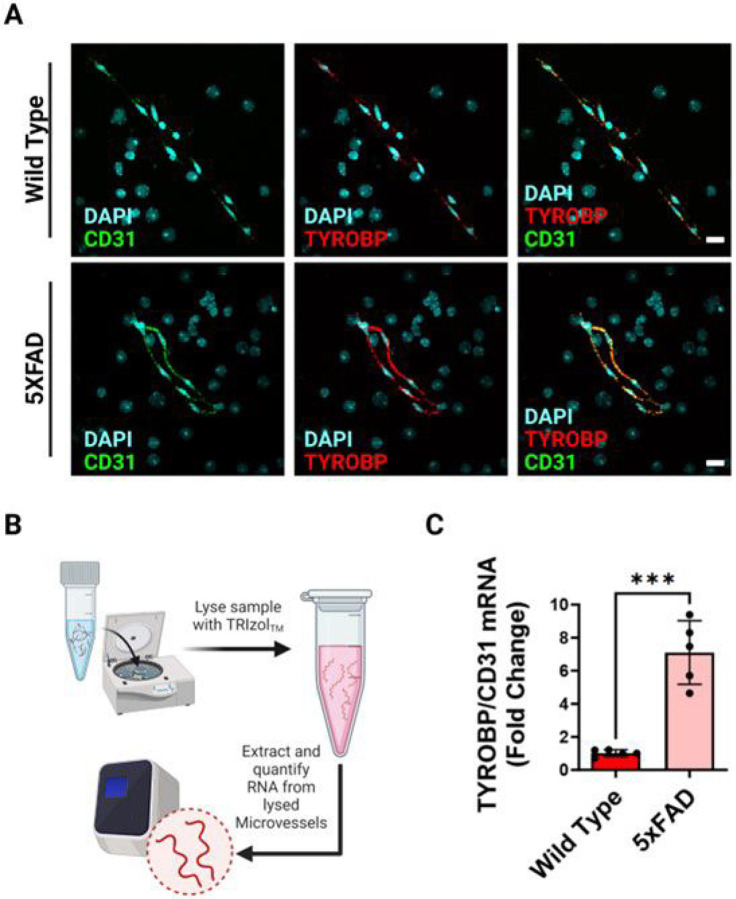
Visualization of TYROBP mRNA in microvessels isolated from 5XFAD mice. (A) The top panel is representative confocal imaging of microvessels after RNAscope isolated from wild type mice. The bottom panel is representative confocal imaging of microvessels after RNAscope isolated from 5XFAD mice. DAPI is visualized in cyan, CD31 mRNA is visualized in green, and TYROBP mRNA is visualized in red, scale bars, 20 μm. (B) Schematic diagram for quantification process via qPCR analysis(C) Quantification of TYROBP mRNA via qPCR analysis of extracted RNA samples from mouse brain microvessels, results were normalized to CD31 mRNA. Graph indicates the mean ± SD from 5 independent experiments. *p < 0.05; **p < 0.01; ***p < 0.001; ****p < 0.0001, n = 5.

A morphological change observed in the 5xFAD model led us to question whether this change translated into any altered signaling pathways. Increased neuroinflammation is a hallmark of AD, however the activated state of the endothelium has not been well characterized in isolated microvessels. We probed for the RNA of TYROBP in the microvasculature to observe how the endothelial cells may become inflammation-activated in a 5xFAD model (Figure 4A). This technique allows for visualizing and quantifying many different RNA probes on isolated microvessels lysed with TRIzol for RNA stability (Figure 4B). To complement traditional quantitative PCR techniques, the visualization of microvessels provides a comprehensive way to visualize RNA levels within biological samples. Total microvessel RNA was isolated from five mice per genotype and quantified for viable RNA via nanodrop. Indeed, the ratio of TYROBP normalized to CD31 RNA levels was significantly increased in the 5xFAD microvessels (Figure 4C). The elevated level of TYROBP tells us that the endothelial cells do become activated and may be important contributors to other neuroinflammatory processes that occur at the BBB.

## Discussion

A viable and robust isolation of the BBB microvasculature is critical for several downstream analyses. However, microvessel isolation protocols often vary in technique and without proper care, can lead to RNA degradation or require a high amount of sensitive biomaterial. Our method yields high quality RNA and is reproducible across different investigators, allowing isolation of all animal blood vessel cell types to study cellular interactions, morphology and signaling changes at the genetic level. This method is highly effective in isolating vascular and vessel-associated cells, such as pericytes, whereas other cellular debris and fat are absent. To the best of our knowledge, RNAscope has never been successfully performed on isolated brain microvessels. Most literature in the field utilizes protein extraction for downstream analysis^7^. Therefore, this protocol offers a novel technique not yet published in the field.

The success of our microvessel isolation is confirmed using a light microscope, and we identify minimal cellular debris, ECs, and pericytes, demonstrating the effectiveness of this isolation procedure. Following microvessel isolation, we probed for the presence of high-quality RNA within the microvessels using the CD31 marker, a universal marker for confirming the presence of blood-brain barrier (BBB) endothelium. We also employ positive and negative control probes to ensure the specificity of their RNA probes to microvascular RNA. Next, we delve into observations in the context of AD. Secondly, we confirmed the loss of pericytes in the 5xFAD mouse model, a phenomenon associated with chronic exposure to amyloid plaques. We used PDGRβ staining to demonstrate a marked decrease in PDGRβ expression in 5xFAD mice, suggesting a significant loss of pericytes. In AD, there is a significant association with pericyte loss or decreased coverage in the microvasculature, contributing to the pathological changes observed in the brain^8^. Pericytes play a crucial role in maintaining the integrity and functionality of the BBB. Their strategic location along capillaries allows them to regulate blood flow, provide vascular stability, and participate in the maintenance of the neurovascular unit. However, in Alzheimer’s, a decline in pericyte density or coverage is evident, compromising the BBB’s structural and functional integrity^9^. This loss of pericytes is linked to increased permeability of the BBB, allowing the infiltration of harmful substances into the brain parenchyma. Additionally, pericyte dysfunction is associated with altered cerebral blood flow, impaired clearance of toxic substances, and the promotion of neuroinflammation, all of which are implicated in the progression of AD. In this protocol, we successfully demonstrate that an AD mouse model does indeed show a decrease in PDGRβ ^+^ nuclei.

While our protocol provides a reproducible and viable RNA technique for many research hypotheses, there is room for protocol adjustment according to the specific research question. A potential confounder is the age of the mice analyzed. The mice used in our experiments were 12 months of age, so chronic exposure to amyloid plaques is predicted to alter the morphology and function of the BBB microvessels. Nevertheless, the blood-brain barrier undergoes alterations during natural aging, distinct from those associated with AD^10^, but the structural changes in the brain vessels (such as a decrease in vessel size) did not demonstrate an influence on the isolation procedure.

Endothelial cells are a vital component that form the lining of blood vessels, maintaining vascular integrity and function. Among the diverse functions they serve, subsets of endothelial cells have been identified to express the tyrosine kinase binding protein (TYROBP), also known as DAP12 or KARAP. TYROBP is a transmembrane signaling adapter protein most associated with immune response modulation and cellular communication by acting as a downstream adaptor for immune receptors such as TREM-1, TREM-2, SIRP1b, CD33, and C3 – many of which are implicated in AD^11^. Interestingly, when expressed in endothelial cells, TYROBP may participate in the regulation of inflammatory processes, leukocyte recruitment, and immune cell interactions within the vascular microenvironment. Notably, the brain vasculature undergoes dynamic remodeling and alterations in response to injury. Moreover, an accumulating body of evidence suggests that brain endothelial cells can become activated due to the recruitment of inflammatory cells^12,13^. We show that the proinflammatory endothelial-activated microvasculature (PEAM) may be associated with TYROBP^+^ endothelium adaptors on the BBB. The activated state of the endothelium is critical to understanding the interplay of signaling adaptors such as TYROBP and circulating ligands, the neurovascular unit, and inflammatory cells in the blood responsible for inducing downstream inflammatory pathways. Indeed, TYROBP has been implicated in the pathogenesis of AD and other neurodegenerative diseases^14^.

This study also explores the impact of AD on the mRNA levels of TYROBP in endothelial cells. TYROBP is associated with neuroinflammation, a hallmark of AD, in microglial cells; however, their involvement with ECs has not been well elucidated. We utilized RNA probes and visualization techniques to quantify TYROBP levels and found a significant increase in TYROBP expression in 5xFAD microvessels compared to the control, indicating that endothelial cells become activated in the disease context. Of note, some studies have demonstrated that TYROBP^+^ ECs do display higher levels of active metabolic pathways and regulation of chemokine pathways^15^. Herein, we successfully show that the endothelium of the BBB also expresses this gene, which may play a role in the recruitment of immune cells to amyloid plaques surrounding vasculature, affecting growth and chemokine signaling of the BBB, and increase the levels of neuroinflammation. Some studies have shown the role of TYROBP^+^ ECs in exacerbating malignant cell progression with a uniquely identifiable metabolic and immunological cellular profile^16^. Additionally, it is thought that microglia and ECs might actively interact through the Icam1-Il2rg and C1qa-Cd93, and microglia might also communicate with each other via Icam1-Itagm, elucidating the importance of this TYROBP pathway in disease-associated endothelial vasculature and the surrounding cellular microenvironment^17^. One of the key roles of TYROBP in AD is its association with microglial activation and the clearance of amyloid-beta plaques, which are a hallmark of AD pathology. Activated microglia, under the influence of TYROBP, can phagocytose and remove these toxic amyloid-beta aggregates. However, in chronic neuroinflammatory conditions, such as those seen in AD, prolonged microglial activation can have detrimental effects, leading to further neurodegeneration.

Here, we present our findings of a microvessel isolation technique for downstream RNAscope analysis. By ensuring their independence from brain parenchyma, isolated brain microvessels can be probed to visualize RNA. This technique involves the digestion of fresh frozen brain hemispheres but can be adapted to the whole brain as well as other species. We also describe the technique for filtration, chemical fixation, followed by the modified application of the RNAscope protocol to visualize and probe for RNA within the microvessels of the brain. It is important to assess the integrity of the microvessels throughout the process. Additionally, this method allows one to assess the overall morphology and signaling pathways that are occurring at the level of the BBB. It is important to assess the integrity and function of the microvessels, particularly since they play a crucial role in controlling cerebral blood flow and maintaining the brain’s homeostatic environment. These findings contribute to our understanding of the role of microvessels in AD and their potential involvement in neuroinflammatory processes at the blood-brain barrier. Utilizing this protocol in scientific research can advance our understanding of the BBB and open new strategies for the effective diagnosis and treatment of neurovascular disorders.

### Limitations:

The success of our protocol depends on high-quality starting tissue samples, specifically fresh or snap frozen tissue before fixation. Microvessel isolation is not possible with fixed tissue, making it essential to prioritize preservation and storage conditions that maintain optimal RNA integrity. Careful tissue collection, handling, and storage are crucial to ensure accurate gene expression analysis and successful microvessel isolation. Implementing proper preservation techniques, such as controlled temperature storage, prevents RNA degradation and guarantees the effectiveness of our protocol.

## Materials and Methods

### Animals

All animals were housed and operated on in an AAALAC accredited facility at the University of Miami. Animal procedures were conducted in accordance with protocols approved by the University of Miami Institutional Animal Care and Use Committee (IACUC) and in accordance with the National Institutes of Health (NIH) guidelines for the care and use of laboratory animals. We mimicked the pattern of Aβ by employing a transgenic mouse model, 5XFAD that is crossbred with a congenic C57B/6J genetic background from Jackson Laboratories (strain #034848). Animals were genotyped with appropriate PCR assays (Transnetyx) resulting in mice hemizygous for the 5xFAD transgenes. The offspring that did not carry the human APP and PSEN1 mutations, were used as wild-type controls. All animals used in the procedures were 12-month-old male and female mice. Both sexes were used for analysis. Mice were maintained in a 12-hour light/dark cycle with ad libitum food and water at room temperature. All studies were approved by the University of Miami Institutional Animal Care and Use Committee, and animals were maintained in accordance with the NIH Animal Welfare guidelines. Reporting of animal data in this manuscript followed recommendations included in the ARRIVE guidelines.

### Microvessel Isolation

Mice were euthanized via 5% isoflurane in oxygen overdose, followed by perfusion with ice cold PBS and rapid decapitation. The brain was meticulously resected from the skull and the brain stem and olfactory bulbs were detached. Additionally, the brain was bisected into hemispheres (0.5 – 1g by weight) and snap frozen in liquid nitrogen to be utilized for the isolation procedure. Following snap freezing brains were stored in −80 degrees Celsius for up to 6 months before microvessel isolation. The brain hemisphere was then placed in a 100 mm petri dish on ice and finely minced by cutting with a fresh blade per brain. Once finely chopped, the tissue pieces were collected and washed with a 2 mL of ice-cold Buffer B and placed into glass homogenizers. The glass homogenizers were fitted with a Teflon pestle and homogenized for 2 minutes (20 up/down motions) until the suspension was uniform. The suspension was transferred through a 300 mm mesh filter into a 50 mL ultra-centrifuge tube. An additional 2ML of Buffer B was used to collect alal the suspension plus 8 mL of 26% Dextran and placed in a rotating incubator (speed 5,800 × g) at 4°C for 20 minutes. Next, the supernatant was aspirated along with the fat layer and pellets of microvessels were resuspended through a cut p1000 pipet tip 10 times with 2 mL of Buffer B. Using an additional 2 mL of Buffer B, the sample was collected and filtered through a 120 mm mesh strainer (Falcon) into 15 mL conicals (Falcon) to remove large undigested fragments. The flacon tubes were spun at 1,500 × g for 10 minutes. The supernatant was removed, and the pellet was resuspended in 200 mL of Buffer B. The resuspended microvessels were visualized using an inverted microscope at 20x magnification. For efficient isolation of vessels and viable RNA, speed is essential and for the entire isolation protocol, temperature changes are limited to a minimum to avoid heat-induced gene activation.

### Microvessel Slide Preparation

This step successfully mounts microvessels on a slide without heat or fixation steps to preserve the integrity of the RNA. This step is adapted from ACD *Cultured Adherent Cell sample Preparation for the RNAscope Mulitplex Fluorescent v2 Assay*. From the 200 mL microvessel suspension, pipette 7 mL on a clean Superfrost charged slide, parallel to the white label on one end. Using a plastic coverslip, gently and evenly apply pressure to smear the suspension across the glass slide. It is important to let the slides air dry for 5 minutes so as not to destroy the RNA. Next, place the prepared slides in a slide staining jar and wash with 1X PBS. Next, transfer the slides into a new jar containing 10% NBF for 30 minutes at room temperature. Following this, repeat with a 1X PBS wash. Finally, dehydrate the slides by transferring into a new slide staining jar containing 50 mL of 50% EtOH > 50 mL of 70% EtOH > 50 mL of 100% EtOH > 50 mL of 100% EtOH for 10 minutes each.

### Fluorescent in situ hybridization: RNAScope

RNA ISH was performed on mouse tissue using the RNAScope Multiplex Fluorescent Detection kit v2 (Advanced Cell Diagnostics, ACD, 323100). The RNAscope experiment was carried out according to the manufacturer’s instructions. In this protocol, RNAs of interest are selectively probed and fluorescently labeled. Probes used in this experiment were: PECAM-1 (ACD, 316721), TYROBP (ACD, 408191-C3), PDGRβ (ACD, 411381-C2), 3-plex positive control probe (ACD, 320881), and 3-plex negative control probe (ACD, 320871). At the end of signal development, slides were stained with fluorophores (ACD, Opal 520, 570, and 590) followed by counterstaining with DAPI. Lastly, sections were mounted using ProLong Gold Antifade Mountant solution (Thermo, P36934). Image acquisition was done on an Olympus FluoView 1200 Laser Scanning Confocal Microscope (Olympus, Center Valley, PA, USA).

### Quantitative Real-Time PCR

Quantitative real-time PCR (qPCR) was performed using Applied Biosystems 7500 system (Applied Biosystems, Foster City, CA). Briefly, mRNA isolation from mouse brain microvessels was performed using the RNeasy mini-kit (Qiagen, Cat# 74104) according to the manufacturer’s instructions. Total RNA was quantified using Nanodrop 2000 (Thermo Fisher Scientific). A total of 100 ng of RNA was used in each reaction. Reverse transcription and qPCR reactions were performed using the qScript XLT 1-Step RT-qPCR Tough Mix (Quantabio, Beverly, MA, USA, Cat #89236–676). The primer probes used for gene amplification by TaqMan Gene Expression Assays (Thermo) were: Mm01242576_m1 (PECAM-1), Mm00449152_m1 (TYROBP). Specificity of qPCR results was established using melting curve assessment, and gene expression fluctuations were determined by the ΔΔCt method, with Ct as the cycle number at threshold. The results were normalized to PECAM-1 expression within the microvessels.

### Image Analysis

All images were analyzed using FIJI. Slides probed for PDGRβ and CD31 were analyzed by manually counting the number of CD31+ nuclei (counterstained with DAPI) as well as the number of PDGRβ + nuclei. Colocalization of RNA fluorescent probes was performed by manual counting. For PDGRβ experiments the number of PDGRβ + cells were taken as a percent fraction of total CD31 mRNA positive nuclei.

### Statistical Analysis

Statistical analyses were performed using GraphPad Prism version 9.5.1 for Windows, GraphPad Software, San Diego, California USA. Each figure panel and legend contain information on statistical tests performed along with sample sizes and p values. *p < 0.05; **p < 0.01; ***p < 0.001; ****p < 0.0001 for all statistical analyses presented in figures. Sample sizes are mentioned in figure legends and indicated as independent experiments (number of animals).

## Data Availability

All data generated or analyzed which support the findings in this study are available within the article.
